# *In vitro* and *in vivo* effects of kisspeptin antagonists p234, p271, p354, and p356 on GPR54 activation

**DOI:** 10.1371/journal.pone.0179156

**Published:** 2017-06-26

**Authors:** C. H. J. Albers-Wolthers, J. de Gier, M. Walen, P. J. S. van Kooten, C. B. Lambalk, P. A. J. Leegwater, B. A. J. Roelen, A. C. Schaefers-Okkens, V. P. M. G. Rutten, R. P. M. Millar, H. S. Kooistra

**Affiliations:** 1 Department of Clinical Sciences of Companion Animals, Faculty of Veterinary Medicine, Utrecht University, Utrecht, The Netherlands; 2 Department of Infectious Diseases and Immunology, Division of Immunology, Faculty of Veterinary Medicine, Utrecht University, Utrecht, The Netherlands; 3 Department of Obstetrics, Gynecology and Reproductive Medicine, VU University Medical Center, Amsterdam, The Netherlands; 4 Department of Farm Animal Health, Faculty of Veterinary Medicine, Utrecht University, Utrecht, The Netherlands; 5 Department of Veterinary Tropical Diseases, Faculty of Veterinary Science, University of Pretoria, South Africa; 6 Centre for Neuroendocrinology and Mammal Research Institute, University of Pretoria, Pretoria, South Africa; 7 Institute for Infectious Diseases and Molecular Medicine, University of Cape Town, Cape Town, South Africa; 8 Centre for Integrative Physiology, University of Edinburgh, Edinburgh, United Kingdom; Universite de Rouen, FRANCE

## Abstract

Kisspeptins (KPs) and their receptor (GPR54 or KiSS1R) play a key-role in regulation of the hypothalamic-pituitary-gonadal axis and are therefore interesting targets for therapeutic interventions in the field of reproductive endocrinology. As dogs show a rapid and robust LH response after the administration of KP10, they can serve as a good animal model for research concerning KP signaling. The aims of the present study were to test the antagonistic properties of KP analogs p234, p271, p354, and p356 *in vitro*, by determining the intracellular Ca^2+^ response of CHEM1 cells that stably express human GPR54, and to study the *in vivo* effects of these peptides on basal plasma LH concentration and the KP10-induced LH response in female dogs. Exposure of the CHEM1 cells to KP-10 resulted in a clear Ca^2+^ response. P234, p271, p354, and p356 did not prevent or lower the KP10-induced Ca^2+^ response. Moreover, the *in vivo* studies in the dogs showed that none of these supposed antagonists lowered the basal plasma LH concentration and none of the peptides lowered the KP10-induced LH response. In conclusion, p234, p271, p354, and p356 had no antagonistic effects *in vitro* nor any effect on basal and kisspeptin-stimulated plasma LH concentration in female dogs.

## Introduction

Kisspeptins (KPs), peptides encoded by the *KiSS1* gene, are key regulators of the hypothalamic-pituitary-gonadal (HPG) axis. The human *KiSS1* gene encodes a peptide of 145 amino acids that can be cleaved into four peptides with a common C-terminal decapeptide terminating in RF-amide: KP54, KP14, KP13, and KP10 [1–3]. These four KPs are the natural ligands for KiSS1R, a G-protein-coupled receptor (also known as GPR54), and have the same binding affinity to the receptor, indicating that the C-terminal 10 amino acid sequence has full intrinsic activity for binding and activation [3–5]. GPR54 is known to be expressed in many mammalian tissues, including brain, pituitary, pancreas, placenta, and smooth muscle of large blood vessels, but the pivotal role of kisspeptin signaling is in reproductive endocrinology [3–7]. Activation of GPR54 by kisspeptins in the hypothalamus results in activation of GnRH neurons and stimulates GnRH secretion [3,8,9]. Kisspeptins and their receptor play a key role in negative and positive feedback effects of gonadal steroids on the hypothalamus. In contrast to kisspeptin neurons, GnRH neurons lack receptors for sex steroids [4,10–12]. Sex steroids stimulate or inhibit the *KiSS1* mRNA concentration in the hypothalamus to mediate positive and negative feedback, respectively [13]. A disruption of kisspeptin signaling, resulting from inactivating mutations of the *KiSS1R* or *KiSS1* gene, results in hypogonadotropic hypogonadism in humans and mice [2,14,15]. Activating mutation of either of these genes is associated with precocious puberty in both man and woman [16–18]. Administration of exogenous KP results in an increase in circulating concentrations of gonadotropins and sex steroids, as has been demonstrated in many species including humans, goats, and dogs [19–22].

The development of kisspeptin antagonists contributed to an improved understanding of the role of kisspeptin in the reproductive system. Roseweir *et al*. [23] developed several KP10 analogues and tested their effect on inositol phosphate (IP) release in Chinese hamster ovarian (CHO) cells stably expressing the human GPR54. Peptide 234 (p234) had the most potent inhibitory effect on IP release *in vitro* [23]. Intracerebroventricular administration of p234 resulted in delayed vaginal opening in rats (an indicator of puberty) and it prevented an increase in the circulating LH concentration when it was co-administrated with KP10. However, p234 alone did not lower the basal plasma LH concentration in intact rats and mice. Additionally, repeated peripheral administration of p271 (p234 with a penetratin tag to allow passage through the blood-brain barrier) could prevent the post-castration rise in circulating LH in male rats and it blunted the KP10-induced rise in plasma LH concentration in mice and rats [23,24]. Furthermore, continuous intracerebroventricular administration of p271 inhibited LH pulses in intact and ovariectomized ewes [25,26].

It is beyond question that KPs and their receptor play a key role in regulation of the HPG axis. These peptides are therefore interesting targets for therapeutic interventions concerning the endocrinological control of reproductive function in mammals. As female dogs exhibit a robust rise in plasma LH, FSH, and estradiol concentrations after peripheral administration of KP10 [22], they represent a good model in which to explore the *in vivo* effects of potential KP agonists and antagonists.

The aims of the present study were to test the antagonistic properties of the kisspeptin antagonists p234, p271, p354, and p356 on Ca^2+^ release *in vitro*, by using CHEM1 cells that stably express the human KiSS1R, and to determine the *in vivo* effect of these peptides on the basal plasma LH concentration and the KP10-induced LH response in female dogs.

## Materials and methods

### Peptides

The following peptides were tested for antagonistic properties on the kisspeptin receptor: p234 ((D-Ala)-Asn-Trp-Asn-Gly-Phe-Gly-(D-Trp)-Arg-Phe-NH2), p271 (Arg-Arg-Met-Lys-Trp-Lys-Lys-Tyr-(D-Ala)-Asn-Trp-Asn-Gly-Phe-Gly-(D-Trp)-Arg-Phe-NH2) [24], p354 ((D-Ala)-Tyr-Asn-Phe-Asn-Gly-Phe-Gly-(D-Trp)-Arg-Phe-NH_2_), and p356 ((D-Ala)-Tyr-Asn-Trp-Asn-Gly-Phe-Gly-(D-Trp)-Lys-Phe-NH_2_). Peptide 354 and p356 are next generation analogs refining p234. They bind and inhibit kisspeptin action on inositol generation in the nanomolar range (unpublished data).

All were produced by the American Peptide Company (APC, Sunnyvale CA, USA) at >95% purity. Human KP10 (hKP10, Tyr-Asn-Trp-Asn-Ser-Phe-Gly-Leu-Arg-Phe-NH2), and canine KP10 (cKP10, Tyr-Asn-Trp-Asn-Val-Phe-Gly-Leu-Arg-Tyr-NH2) were also purchased from APC at >95% purity. All peptides were dissolved in Aquadest (MilliQ®, Millipore BV, t]he Netherlands) to a stock concentration of 10^−4^ M. The stock solutions were further diluted to the needed concentrations, with HBSS+++ (Hank’s Balanced Salt Solution (HBSS, Gibco, Life Technologies, the Netherlands) supplemented with 10 mM HEPES (Gibco, Life Technologies, the Netherlands), 0.05% w/v Bovine Serum Albumin (BSA, A7030, Sigma Aldrich, the Netherlands) and 2.5 mM probenecid (P8761, Sigma Aldrich, the Netherlands, using 500 mM stock freshly prepared in 1 M NaOH). The peptides for *in vivo* use, were dissolved in dimethylsulfoxide (DMSO) and water for injection to achieve a concentration of 200 μg peptide antagonist per ml. Final solutions contained 2% DMSO or less. The solution was then divided into 10 ml portions, stored at -20°C, and thawed within one hour before use.

### Flow cytometric calcium flux assay

*In vitro* analyses of the effects of kisspeptin and kisspeptin antagonists, were performed using HTS032C cells (rat hematopoietic CHEM1 cells expressing endogenous Gα15 and, stably expressing the human GPR54 receptor (Millipore BV, the Netherlands), CHEM1-GPR54. Cells were counted and suspended in HBSS+++ at a concentration of 5*10^6^ cells/ml. To load the cells with the fluorescent dye ester they were incubated in HBSS+++ adjusted to a concentration of 2 μM Fluo-3 AM (Molecular Probes, Life Technologies, the Netherlands) for 1h at 37°C under agitation in the dark. After incubation, the cells were washed by adding HBSS+++ to a volume of 10 ml followed by centrifugation (5 min, 150 x g) to remove excess of Fluo-3AM. The supernatant was discarded, the cell pellet was resuspended to 10^6^ cells/ml in HBSS+++, and 225 μl labeled cells (10^6^ cells/ml HBSS+++) were pipetted into 5 ml high optical clarity polystyrene Round-Bottom Tubes (Falcon 352052, Becton Dickinson, the Netherlands). The cells were aspirated by the flow cytometer (FacsCalibur, Becton Dickinson, the Netherlands) and their fluorescence was assessed by using the FL1 channel at 10 ms intervals during a total of 60 seconds, according to the following procedure. During the first 10 seconds the baseline fluorescence due to binding of spontaneously released Ca2+ to the ester into the Fluo-3–Ca^2+-^complex was measured. Thereafter the tube was removed briefly and 25 μl peptide (see below) was added, after which aspiration and analysis continued. For statistical analyses fluorescent data recorded during the time interval 30–60 seconds after initiation of measurement were used.

### Calcium responses of CHEM1-GPR54 cells induced by human and canine KP10

**Fig 1:**
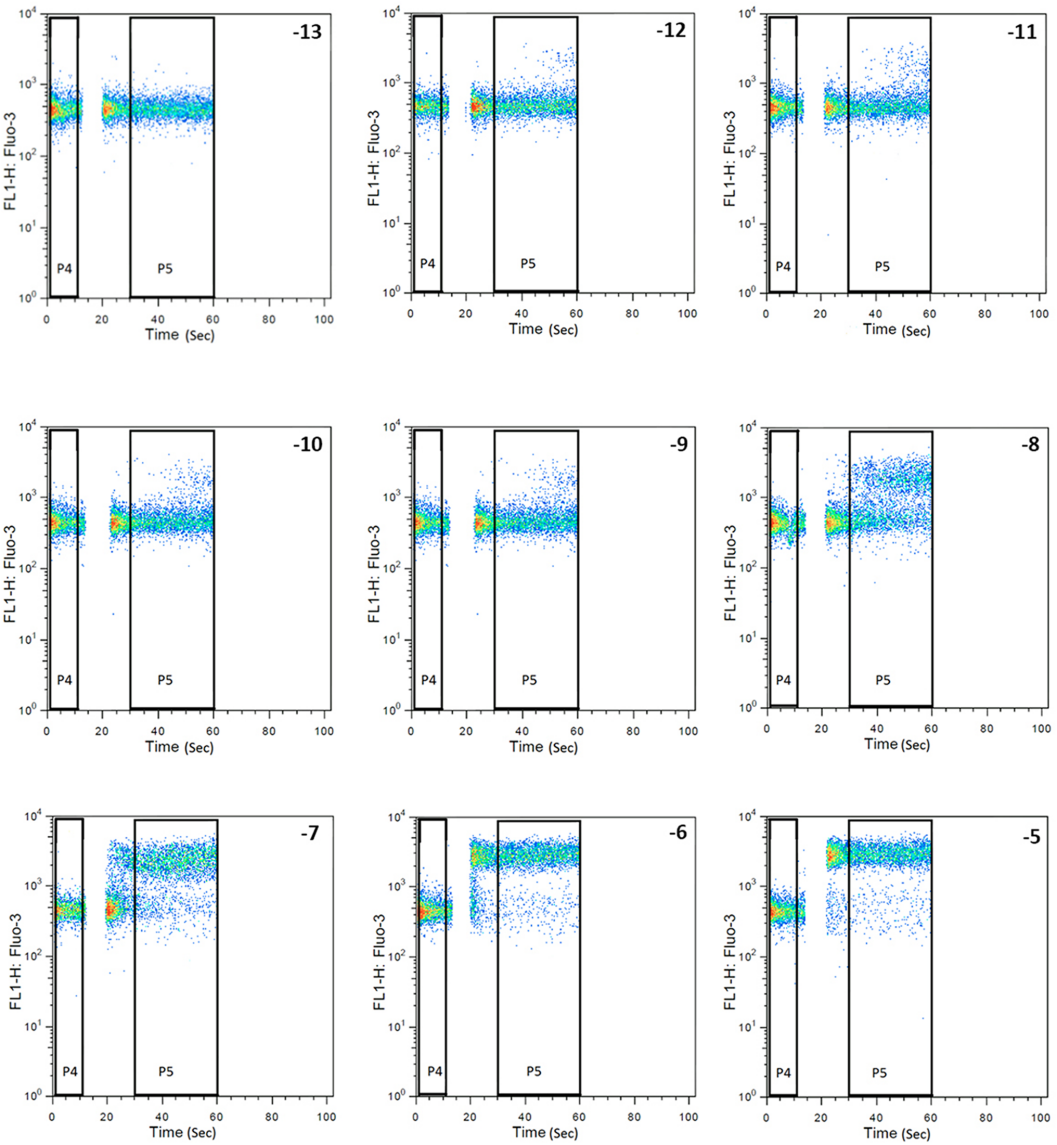
FACS analysis of CHEM1-GPR54 cells before and after addition of human KP10. Representative results of FACS analysis of the calcium flux after addition of different concentrations (10^−13^ to 10^−5^ M) of human KP10 to CHEM1-GPR54. P4 (0-10S) and P5 (30-60S) were the time frames in which the fluorescence intensity of cells was registered and the means of baseline and stimulated levels were calculated. Human KP10 was added during the gap between P4 and P5, 10–30 seconds after the start of the FACS analysis. The concentrations of human KP10 are indicated in the upper right corner of each plot.

In order to construct a dose-response curve, CHEM1 cells were stimulated with 10^−13^ M to 10^−5^ M (10-fold dilution steps) of hKP10 and cKP10. Separate tubes were used for each concentration of KP10 starting with the lowest concentration (10^−13^ M) to prevent contamination by residuals in the internal system of the flow-cytometer. In negative control tubes, 25μl HBSS+++ was added to the cells after 10 seconds of baseline fluorescence measurement. Experiments were performed 3 times. Representative flow cytometry results are depicted in Fig 1.

### Antagonists impact on the human and canine KP10 induced calcium responses of CHEM1-GPR54 cells

**Fig 2:**
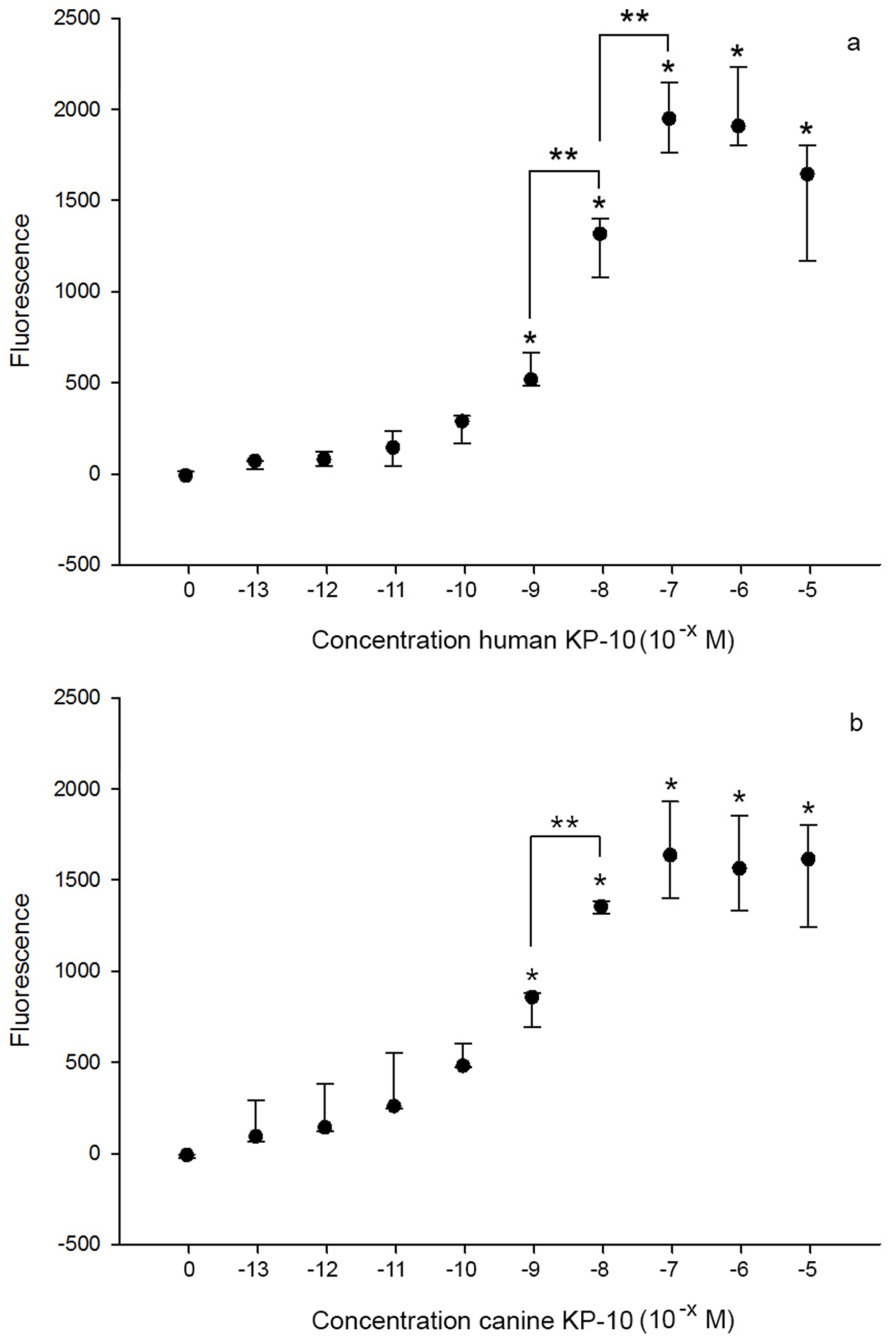
Dose response curves of human and canine KP10 on CHEM1-GPR54 cells. Dose response curves of Ca^2+^ responses (median fluorescence; n = 3) of CHEM1-GPR54 cells for concentration ranges of human (a) and canine (b) KP10. The error bars represents the range (min and max). * indicates a significant difference (P<0.05) compared to the control (no KP10). ** indicates a significant difference between two consecutive concentrations.

Initially the concentrations 10^−13^ M to 10^−5^ M of the four antagonists (p234, p271, p354, and p356) were tested in the same manner as human and canine KP10 concentrations to determine whether these peptides gave rise to an intrinsic calcium flux. We then assessed whether these antagonists could compete with KP10 for its receptor. To test the effect of pre-incubation of the cells with the antagonists (in increasing concentrations from 10^−13^ M to 10^−5^ M) on the KP10 induced calcium response, the concentration of KP10 that resulted in a sub-maximum calcium response was used. For both human and canine KP10, the concentration of 10^−8^ M KP10 was located on the linear part of the sigmoid dose-response curve (Fig 2A and 2B) and this concentration was considered to result in a sub-maximum calcium response. For this purpose 25 μl quantities of the antagonists were added to 200 μl of Fluo-3 AM labelled cells (10^6^ cells/ml) and incubated for 15 min. Next, after baseline measurements of fluorescence for 10 seconds in the flowcytometer, the tubes were briefly removed for the addition of 25 μl of 10^−8^ M KP10 and the measurement was continued. As a control, 10^−8^ KP10 was added to 200 μl cells with 25 μl HBSS+++, that had not been exposed to an antagonist. Peptides p354 and p356 were tested three times with both human and canine KP10; while peptides p234 and p271 were tested three times with human KP10 and once with canine KP10.

### Animals

Thirteen adult anestrous Beagle bitches were used for this study. Six dogs with a median age of 89 months (range 43–122 months) were used as control group, receiving cKP10 without an antagonist. Eight dogs with a median age of 52 months (range 31–116 months) were used for the antagonist experiments. One dog was first used in the control group and 12 months later for the antagonist experiments. Of the eight dogs used for the antagonist experiments, four received p271, p354, and p356. In two of the eight dogs both p354 and p356 were tested, while in the remaining two dogs only p271 was tested, in order to test every antagonist in 6 dogs. The time between testing of different kisspeptin antagonists was at least 5 days (range 5–15 days) to ensure that there were no peptide residues. Kisspeptin antagonist p234 per se was not tested *in vivo* because p271 is in fact p234 with a penetratin tag to enhance passage across the blood-brain barrier, as has been described for use with peripheral administration [24].

All dogs were born and raised in the Department of Clinical Sciences of Companion Animals and were accustomed to the laboratory environment and procedures such as the collection of blood. They were housed in pairs in indoor/outdoor runs, fed a standard commercial dog food once daily, and provided with water ad libitum.

All dogs were examined thrice weekly for swelling of the vulva and serosanguineous vaginal discharge, signifying the onset of proestrus [27]. Plasma progesterone concentration was measured thrice weekly from the start of proestrus until it exceeded 13–16 nmol/l, at which time ovulation is assumed to occur [28–30]. Anestrus was defined as the period from 100 days after ovulation to the onset of proestrus, as indicated by vulvar swelling and serosanguineous discharge.

### Experimental design of in vivo study

Kisspeptin antagonists (50 μg/kg/h) were administered continuously for three hours via a catheter in the cephalic vein. Two hours after the start of the infusion, a single bolus of cKP10 (0.5 μg/kg) was administered intravenously. Blood samples were collected from the jugular vein by venipuncture at -40 min and 0 min before the antagonist infusion, and then every 30 min for two hours. After the administration of cKP10, blood samples were collected at 10, 20, 40, and 60 min (i.e., 130, 140, 160, and 180 min after the start of the antagonist infusion).

As a control, KP stimulation tests (without the administration of a kisspeptin antagonist) were performed in six dogs. Blood samples were collected from the jugular vein by venipuncture at -40 min and 0 min before, and then at 10, 20, 40, and 60 min after administration of cKP10 (0.5 μg/kg). Plasma LH concentrations were measured in all blood samples.

### Hormone assays

Plasma progesterone concentrations were measured thrice weekly during the follicular phase to determine the ovulation period, using a I-125 radioimmunoassay previously validated for ovulation timing [31]. The intra- and interassay coefficients of variation (CVs) were 6 and 10.8%, respectively, and the limit of quantitation was 0.13 nmol/l.

Plasma LH concentrations were measured with a heterologous radioimmunoassay validated for the dog, as described previously [32,33]. The intra- and interassay CVs for values above 0.5 μg/l were 2.3 and 10.5%, respectively, and the limit of quantitation was 0.3 μg/l.

### Data analysis

The effects of the various peptides on CHEM1-PGR54 cells were assessed by the Flowcytometric calcium flux assay in order to construct a dose response curve for human and canine KP10, to select an optimal dose of the KP10s for in vitro antagonist testing, and to determine whether different concentrations of the antagonists (p234, p271, p354, and p356) resulted in an intrinsic calcium response. The differences in the mean fluorescence intensity (as a measure for the calcium response) before and after addition of the peptide were calculated and used for statistical analysis using SPSS® for Windows, version 22 (SPSS Inc., Chicago, USA). A multivariate general linear model was set up to analyze differences in calcium response between the different concentrations, with Dunnett’s test as a post hoc procedure for the antagonists and Bonferroni’s test for the agonists. A probability-probability plot of all used peptides showed that the residuals of the ANOVA were normally distributed.

To analyze the effects of different concentrations of the KP antagonists on the calcium response due to KP10 addition, the differences in fluorescence before and after KP10 were calculated (the calcium response). The calcium response ratios were calculated as the calcium response of the CHEM1-GPR54 cells pre-incubated with different concentrations of KP antagonist and the control (the calcium response of the cells without KP antagonist). These calcium response ratios were log transformed and used for further analysis by univariate analysis of variance, with the Dunnett’s test as post hoc test. The calcium response ratios were set as dependent variables and the different concentrations of the antagonists and the two types of KP10 were used as fixed factors. The residuals were normally distributed.

In both groups of dogs (experimental and control), the basal plasma LH concentration was calculated for each dog as the mean of the values at –40 and 0 min before cKP10 or antagonist administration. All LH values that exceeded the mean of all basal samples plus 3 SD were attributed to pulsatile secretion and were treated as outliers and excluded from statistical analysis. Log transformation of plasma LH concentrations was used because data were not normally distributed. A linear mixed model was used to analyze differences in plasma LH concentrations before and after KP10, with or without a kisspeptin antagonist (group effects: p271, p354, p356, and controls), with Bonferroni correction. P<0.05 was considered significant.

### Ethics of experimentation

This study was approved by the Ethical Committee of Utrecht University (DEC III.08.076) conform Dutch legislation.

## Results

### Calcium responses of CHEM1-GPR54 cells induced by human and canine KP10

The addition of KP10 to the CHEM1 cells containing high endogenous levels of the G protein Gα15 and expressing human GPR54 resulted in a calcium response, the mean fluorescence being dependent on the number of cells responding to KP10 (Fig 1). The dose-response curves for human and canine KP10 are shown in Fig 2A and 2B, respectively. The EC50 of human and canine KP10 is 1.2 nM and 8 nM, respectively. Since for both human and canine KP10 the concentration of 10^−8^ M KP10 was located on the linear part of the sigmoid dose-response curve, this concentration was used to test the effects of the antagonists on the KP10-induced calcium response of the CHEM1 cells.

### Calcium responses of CHEM1-GPR54 cells induced by KP antagonists

**Fig 3:**
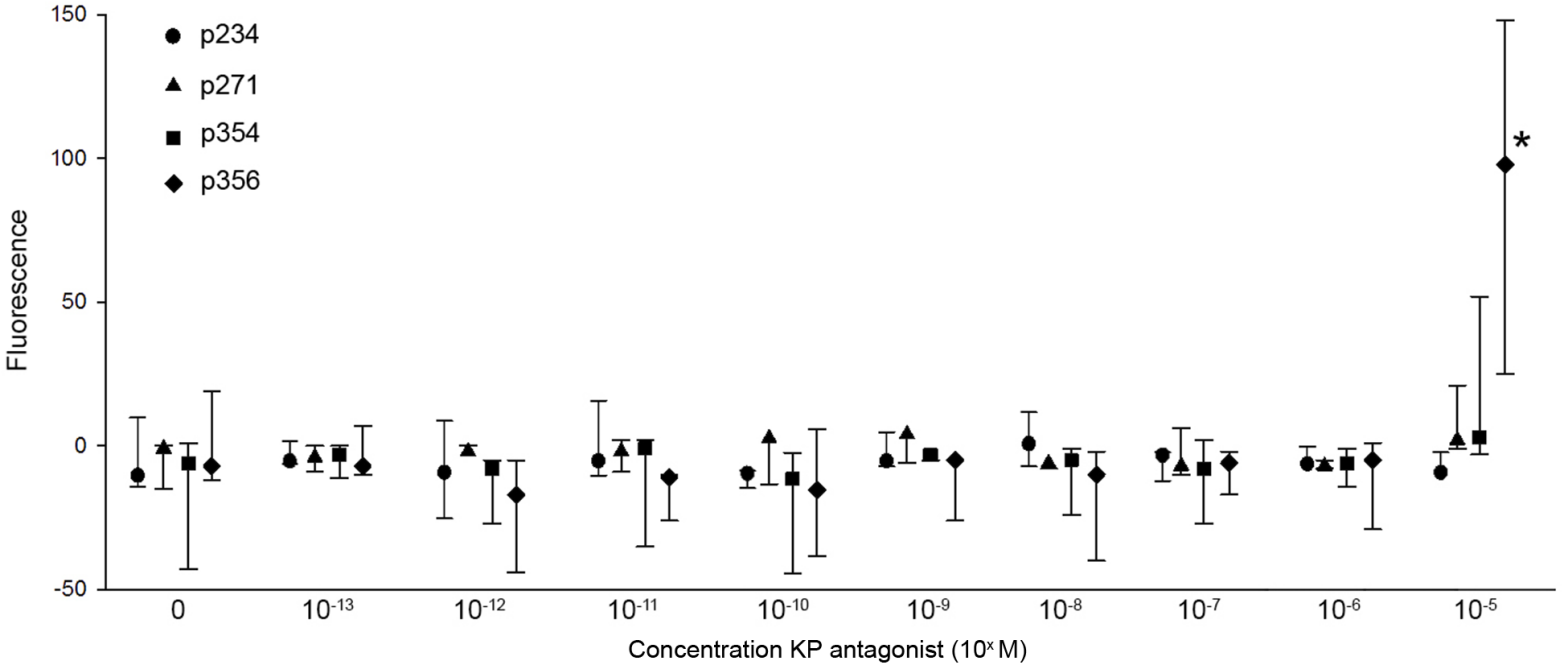
Dose response curves of p234, p271, p354 and p356 on CHEM1-GPR54 cells. Dose response curve of Ca^2+^ responses (median fluorescence; n = 3) of CHEM1-GPR54 cells concentrations ranges of kisspeptin antagonist p234, p271, p354, and p356. The error bars represents the range (min and max). * indicates a significant difference (P<0.05) from control (zero).

There was no significant intrinsic calcium response in the CHEM1-GPR54 cells at any of the tested concentrations (10^−13^ to 10^−5^ M) of p234, p271, and p254. Only at the highest concentration (10^−5^ M) of p356 a clear calcium response (P = 0.001) was observed (Fig 3).

### Antagonists impact on the human and canine KP10 induced calcium responses of CHEM1-GPR54 cells

**Fig 4:**
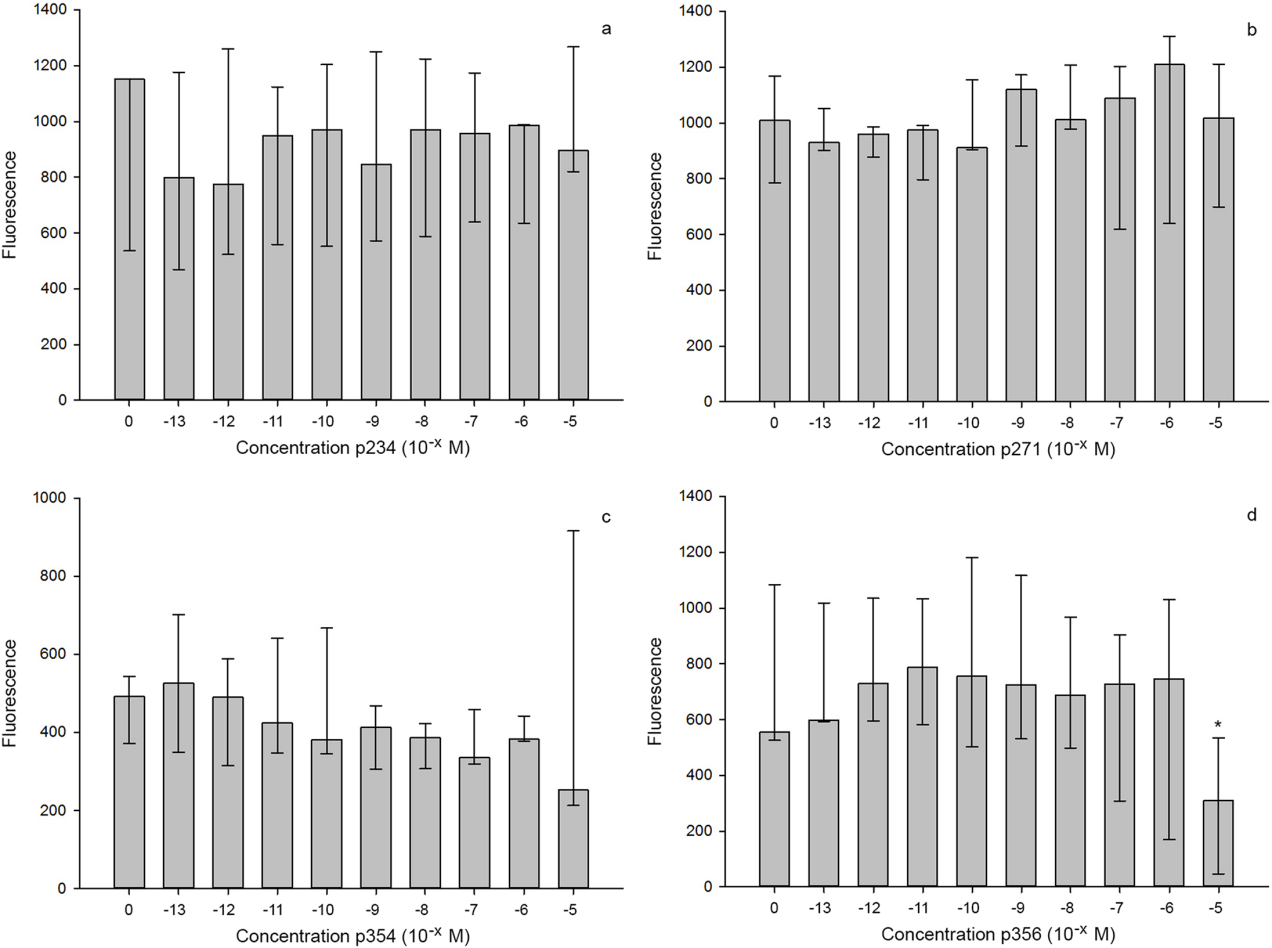
Calcium responses of CHEM1-GPR54 cells to human KP10 after incubation with different concentrations of p234, p271, p354 and p356. The median (n = 3) Ca^2+^ response of CHEM1-GPR54 cells to the addition of 10^−8^ M human KP10 and 10^−13^–10^−5^ M of p234 (a), p271 (b), p354 (c), and p356 (d). The error bars represents the range (min and max). * indicates a significant difference from control (no antagonist).

The difference in the calcium responses between human and canine KP10 was not significant (P = 0.09). Fig 4 shows the calcium responses due to addition of human KP10 to CHEM 1-GPR54 cells after pre-incubation at different concentrations of the different antagonists. The differences in the calcium response ratio between the controls and all concentrations of antagonists p354, p234, and p271 were not significant (P = 1.00). The calcium response ratio of p356 was smaller than the negative control only at the highest concentration of 10^−5^ M (P<0.001).

### Effects of the antagonists on plasma LH concentration in female dogs

**Fig 5:**
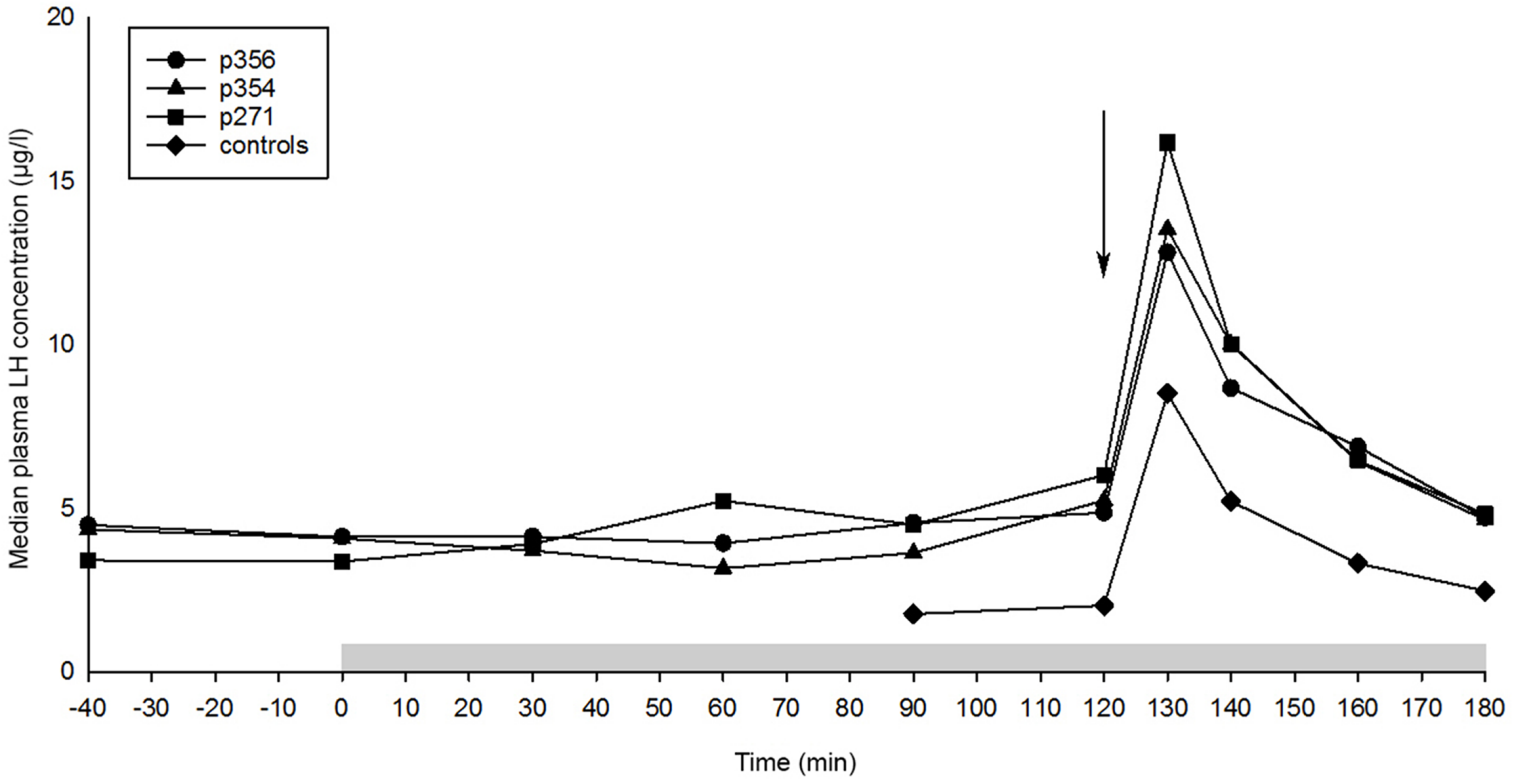
The plasma LH concentration during kisspeptin antagonist infusion and single canine KP10 administration. The median plasma LH concentration (μg/L) during continuous infusion of 50 μg/kg/h of different KP antagonists and the controls. n = 6 dogs per group. At t = 120 min 0.5 μg canine KP10/kg was administered intravenously. The antagonist infusion started at t = 0 min and lasted until t = 180 min (grey bar). The arrow indicates the time of cKP10 administration.

In order to understand how the kisspeptin antagonists would affect reproduction, anestrous female dogs were exposed to the antagonists. The median plasma LH concentrations before and during the kisspeptin antagonist infusion and before and after canine KP10 administration are shown in Table 1. The continuous intravenous administration of the antagonists (p271, p354, and p356) did not cause significant alterations in basal plasma LH concentrations (Fig 5). Administration of cKP10 resulted in a significant increase (P<0.001) in plasma LH concentration in all bitches, in all groups. In all groups plasma LH concentration was maximum at 10 min after cKP10 administration and then decreased to basal levels at 60 min. The median maximum increment in plasma LH concentration was 12.2 μg/L (range 4.7–18.2 μg/L) during p271 administration; 8.1 μg/L (range 5.0–12.5 μg/L) during p354 administration; 8.1 μg/L (range 6.3–18.3 μg/L) during p356 administration, and 6.5 μg/L (range 2.8–24.7 μg/L) in the control group. Differences in plasma LH concentration between the experimental groups and the controls before and after cKP10 administration were not significant (P = 0.20).

**Table 1:** Median (and range) plasma LH concentrations (μg/L). * = min. after cKP10 administration.

Group	Basal	t = 30	t = 60	t = 90	t = 120	t = 130 / t = 10*	t = 140 / t = 20*	t = 160 / t = 40*	t = 180 / t = 60*
**controls**	1.8 (1.3–5.5)	n/a	n/a	n/a	n/a	8.5 (5.1–26.8)	5.2 (4.0–11.9)	3.3 (2.5–5.2)	2.5 (1.3–3.5)
**p354**	4.2 (2.8–6.7)	3.7 (2.1–13.1)	3.2 (2.2–7.2)	3.6 (3.0–6.3)	5.2 (2.6–15.7)	13.5 (8.4–20.3)	10.0 (5.5–13.0)	6.4 (4.7–9.1)	4.7 (3.7–9.9)
**p356**	4.3 (2.8–6.0)	4.1 (2.4–12.8)	3.9 (2.2–7.7)	4.5 (2.8–6.7)	4.9 (2.1–10.4)	12.8 (9.1–23.1)	8.7 (7.1–13.4)	6.9 (3.7–12.8)	4.7 (3.6–12.8)
**p271**	4.0 (2.9–5.9)	3.9 (2.2–5.4)	5.2 (2.5–10.0)	4.5 (2.8–6.3)	6.0 (3.2–7.5)	16.2 (9.3–24.1)	10.0 (5.7–12.6)	6.4 (4.8–8.8)	4.8 (3.5–7.3)

Median and range of plasma LH concentrations before and during antagonist p271, p354, and p356 infusion. At t = 120 min canine KP10 (0.5 μg/kg) was administered intravenously. n = 6 dogs per group.

## Discussion

In the present study, the antagonistic properties of four peptides (p234, p271, p354, and p356) were studied *in vitro* by using CHEM1 cells that stably express human GPR54 and *in vivo* by using anestrous female dogs as a model to test antagonistic properties of these peptides on basal and KP10-stimulated plasma LH concentrations. Since KP signaling is essential for normal functioning of the HPG-axis, KP and its receptor are interesting targets for therapeutic interventions concerning reproductive function, such as induction of ovulation or contraception. The dog appeared to be particularly sensitive to exogenous KP10 and thus a good model for KP inhibition studies such as testing antagonistic effects of KP analogs [22]. KP signaling is also an interesting target for investigation of nonsurgical contraception in dogs.

Pet overpopulation and stray dogs cause major problems world-wide. A reduction in the number of stray dogs will result in a decreased risk of dog bites and the incidence of rabies in humans [34]. At present the only effective, reliable, and permanent method for contraception in this species is gonadectomy. This procedure requires specially trained people, is time consuming and costly, is traumatic and presents surgical and anesthetic risks for the animal [35]. A nonsurgical method that results in lifelong contraception after a single treatment would be of great value for sterilization of a large portion of the feral population within a relatively short time. The present study is therefore of importance for both human and canine medicine.

Canine and human KP10 differ at two amino acid positions [22], but these differences do not affect receptor activation, as both peptides resulted in similar calcium responses. The peptide antagonists are KP10 analogs with substitutions at several positions, which should result in good receptor binding affinity but reduced receptor activation [36]. None of the peptides tested in the present study prevented or inhibited the KP10-stimulated calcium response and thus receptor activation. This is in contrast to the findings of Roseweir et al. [23], who showed that p234 resulted in a 93% inhibition of KP10-induced inositol phosphate (IP) production in CHO cells that express GPR54. As both IP and Ca^2+^ can be used as a measure of receptor activation, the use of different methods in studying the antagonistic properties of p234 is not likely to be the cause of the contradictory results between the study of Roseweir et al. [23] and the present study. All peptides used in the present study were from the same batch, but different from the batch used by Roseweir et al. [23], which may explain different results. However, all of the peptides used in the present study had >95% purity and in preliminary studies (unpublished data) other batches of the peptides were used with similar results as described here. It is therefore very unlikely that the lack of antagonistic properties of p234 was due to a poor batch. Poor solubility of the peptides could also result in a lack of effects but all were found to be quickly and apparently completely dissolved in the protocol used in this study and there were no visible residues in the vials.

Although several studies have shown that some of the used antagonists, particularly p234, have antagonistic properties on the KiSS1R *in vitro (*and *ex vivo)* [23,36–39] and *in vivo* in mice [23], rats [24,40,41], sheep [26,42,43], monkeys [44], and fish [45], the present study did not demonstrate antagonist activity of these peptides. The failure of the antagonists to exhibit activity in the current study therefore presents a conundrum. Since there are differences in the sequence of the canine KP receptor and KP10 it is possible that the antagonists bind poorly even though KP10 itself appears to bind well based on the efficacious stimulation of LH. We are unable to make a judgement on these possibilities in the absence of pharmacokinetic and binding studies which were not conducted in the current study.

In the present study, CHEM1-GPR54 cells in suspension were used, in contrast to the study of Roseweir et al. [23], where adherent CHO cells were used for testing the antagonistic effects of KP antagonists (including p234). In order to rule out that the effects of KP analogs are influenced by the method of cell culture, preliminary studies has been performed by using adherent CHEM1-GPR54 cells and a calcium flux assay (unpublished data). In those preliminary studies, human- and canine KP10 induced a clear calcium response, but this response could not be inhibited by p234 or p271. The amount of repeats was too low to perform statistics and to publish. A major advantage of flow cytometry with cells in suspension is that it is less time-consuming and therefore it is possible to retrieve a large quantity of data in a relatively short time. The fact that the adherent cell line showed similar effects of both the KP agonists and p234 on CHEM1-GPR54 cells as the results in the present study, a lack of antagonistic effect appears not due to the use of cells in suspension.

Since peptide antagonists must compete for receptor (GPR54) occupation with the agonist, KP10, a superior binding affinity and a more rapid receptor on-rate of KP10 might hide antagonistic effects. Therefore, the cells were incubated with the antagonist for 15 min before the agonist, KP10, was added. Furthermore, a wide range of concentrations of the antagonists were tested to determine whether they could compete with KP10. The concentration of 10^−8^ hKP10 is the same as that used in the study of Roseweir et al. [23] and it resulted in a submaximal calcium response and should be ideal for the purpose of competitive binding studies.

Addition of one of the peptides, p356, resulted in an intrinsic calcium response at the highest concentration (10^−5^ M p356). The difference in the intracellular calcium concentration before and after KP10 addition was therefore smaller. This explains the significantly lower KP10-induced calcium response ratio than observed for other concentrations and other peptides, which however was not due to an antagonistic effect of p356.

In keeping with the *in vitro* results of the present study, none of the supposed antagonist peptides had an antagonistic effect *in vivo* in anestrous bitches. The kisspeptin antagonists were administered intravenously in a dose of 50 μg/kg/h, and had no effect on either the basal plasma LH concentration during the continuous administration or on the KP10-induced LH response. These findings are in line with a previous study in female dogs [46]. In contrast, antagonistic effects at doses comparable to those used in the present study have been reported in rats. Adult male rats received an intraperitoneal injection of 5 nmol p271 every hour for three hours. The last dose of p271 was combined with an intraperitoneal or intracerebroventricular injection of KP10. The KP10-induced LH response was absent after intraperitoneal injection and was blunted after intracerebroventricular administration [24]. These results may suggest species-related differences in the effects of KP antagonists.

Other studies with p271 in adult castrated male dogs and adult anestrous bitches conducted at our facilities, using an experimental design comparable to that in the present study, were performed with doses of up to 600 μg/kg/h. These doses also had no effect on plasma LH concentration in the castrated male dogs or on the KP10-induced LH response in the female dogs (unpublished data). It is therefore unlikely that the lack of antagonistic effects is related to the dose of 50 μg/kg/h used in the present study.

Sensitivity to exogenous KP10 differs according to the stage of the reproductive cycle, as has been shown in rats, ewes, bitches, and women [19,46–48]. In the bitch, the highest KP10-induced LH response was obtained during anestrus and the lowest was during the follicular phase [46]. It is possible that the effects of a KP antagonist also differ among the cycle stages. However, p271 administration to bitches during different stages of the estrous cycle (including anestrus, the follicular phase, and during two stages of the luteal phase) did not result in a decrease in the basal plasma LH concentration nor did it lower the KP10-induced LH response [46]. Consequently, it appears unlikely that the other peptides (p234, 354, and 356) will have antagonistic effects in dogs during stages of the estrous cycle other than anestrus.

Our findings therefore suggest caution in utilisation of these antagonists across species *in vivo* and different signaling monitoring systems. It is imperative to thoroughly characterise binding kinetics and signaling of antagonists at the receptor of interest, metabolic clearance rates and modes of administration. The current study is therefore of value in alerting researchers to this.

In conclusion, the KP antagonists p234, p271, p354, and p356 did not have antagonistic effects on CHEM1-GPR54 cells, nor did the peptides p271, p354, and p356 have antagonistic effects in anestrous dogs. Based on the effects p356 on the CHEM1-GPR54 cells, it can be concluded that this peptide is a partial agonist rather than an antagonist.

## Supporting information

S1 Dataset CHEM1-GPR54 fluorescence before and after adition of canine KP10, human KP-10, p234, p271, p354 and p356. (PDF)

S2 Dataset Plasma LH concentration in anestrous bitches before, during and after canine KP10, p271, p354 and p356 administration. (PDF)
