# Supplementary material for: In vitro and in vivo effects of kisspeptin antagonists p234, p271, p354, and p356 on GPR54 activation
Source: PLoS One. 2017 Jun 26;12(6):e0179156. doi: 10.1371/journal.pone.0179156 (PMC5484485; doi:10.1371/journal.pone.0179156)
Supplement: S1 Dataset — (PDF) [file pone.0179156.s001.pdf]

S1. FACS analysis of CHEM1-GPR54 cells. A: Concentration response curve. Mean fluorescence of CHEM1-GPR54 cells before (p4) and after (p5) addition of different concentrations ($1*10^{-13}$ - $1*10^{-5}$ M) kisspeptin analogs: human KP10 (hKP10), canine KP10 (cKP10), p234, p271, p354 and p356. B: Mean fluorescence of CHEM1-GPR54 cells incubated with different concentrations ($1*10^{-13}$ - $1*10^{-5}$ M) of kisspeptin antagonists p234, p271, p354 or p356, before (p4) and after (p5) stimulation with $1*10^{-8}$M hKP10 or cKP10.

A

Repeat		P4	P5
1	control	395	400
	hKP10 10-13	368	438
	hKP10 10-12	368	416
	hKP10 10-11	364	452
	hP10 10-10	398	519
	hKP10 10-9	392	677
	hKP10 10-8	389	1338
	hKP10 10-7	385	2287
	hKP10 10-6	387	2214
	h10-5 hKP10	332	1146
		P4	P5
2	control	456	463
	hKP10 10-13	476	498
	hKP10 10-12	467	504
	hKP10 10-11	459	497
	hKP10 10-10	476	635
	hKP10 10-9	440	916
	hKP10 10-8	462	1857
	hKP10 10-7	463	2603
	hKP10 10-6	446	2675
	hKP10 10-5	455	2249
		P4	P5
3	control	471	456
	hKP10 10-13	463	528
	hKP10 10-12	466	580
	hKP10 10-11	474	702
	hKP10 10-10	468	780
	hKP10 10-9	466	1127
	hKP10 10-8	453	1528
	hKP10 10-7	463	2407
	hKP10 10-6	462	2365
	hKP10 10-5	444	1609

Repeat		P4	P5
1	control	336	333
	cKP10 10-13	338	436
	cKP10 10-12	337	485
	cKP10 10-11	333	586
	cKP10 10-10	335	815
	cKP10 10-9	335	1036
	cKP10 10-8	334	1695
	cKP10 10-7	334	1976
	cKP10 10-6	334	1904
	cKP10 10-5	333	1954
		P4	P5
2	control	330	330
	cKP10 10-13	331	629
	cKP10 10-12	333	723
	cKP10 10-11	322	882
	cKP10 10-10	330	936
	cKP10 10-9	330	1216
	cKP10 10-8	327	1651
	cKP10 10-7	328	1736
	cKP10 10-6	325	1664
	cKP10 10-5	325	1574
		P4	P5
3	control	496	476
	cKP10 10-13	497	568
	cKP10 10-12	502	631
	cKP10 10-11	498	763
	cKP10 10-10	490	976
	cKP10 10-9	500	1364
	cKP10 10-8	506	1897
	cKP10 10-7	500	2436
	cKP10 10-6	491	2350
	cKP10 10-5	499	2307

		P4	P5
1	control	440	429
	P234 10-13	440	433
	P234 10-12	447	421
	P234 10-11	436	451
	P234 10-10	444	429
	P234 10-9	437	441
	P234 10-8	439	450
	P234 10-7	457	444
	P234 10-6	459	452
	P234 10-5	454	444

		P4	P5
2	control	422	431
	P234 10-13	437	431
	P234 10-12	437	445
	P234 10-11	440	429
	P234 10-10	439	429
	P234 10-9	434	428
	P234 10-8	434	434
	P234 10-7	433	430
	P234 10-6	432	425
	P234 10-5	419	416

		P4	P5
3	control	471	456
	P234 10-13	445	446
	P234 10-12	451	441
	P234 10-11	442	436
	P234 10-10	441	432
	P234 10-9	442	434
	P234 10-8	441	433
	P234 10-7	439	435
	P234 10-6	439	438
	P234 10-5	437	427

		P4	P5
1	control	289	246
	p354 10-13	250	239
	p354 10-12	275	248
	p354 10-11	284	249
	p354 10-10	295	251
	p354 10-9	266	263
	p354 10-8	255	231
	p354 10-7	278	251
	p354 10-6	323	309
	p354 10-5	316	368

		P4	P5
1	control	369	369
	P271 10-13	375	366
	P271 10-12	367	367
	P271 10-11	375	373
	P271 10-10	362	365
	P271 10-9	365	369
	P271 10-8	369	361
	P271 10-7	359	365
	P271 10-6	365	357
	P271 10-5	358	379

		P4	P5
2	control	383	382
	P271 10-13	384	380
	P271 10-12	382	380
	P271 10-11	381	383
	P271 10-10	380	383
	P271 10-9	384	388
	P271 10-8	380	374
	P271 10-7	376	369
	P271 10-6	375	368
	P271 10-5	372	371

		P4	P5
3	control	471	456
	P271 10-13	449	449
	P271 10-12	452	450
	P271 10-11	459	450
	P271 10-10	456	443
	P271 10-9	456	450
	P271 10-8	457	451
	P271 10-7	455	445
	P271 10-6	448	443
	P271 10-5	459	461

		p4	p5
1	control	294	282
	p356 10-13	305	298
	p356 10-12	316	272
	p356 10-11	320	294
	p356 10-10	314	276
	p356 10-9	303	277
	p356 10-8	309	269
	p356 10-7	296	279
	p356 10-6	316	287
	p356 10-5	292	440

		P4	P5			P4	P5
2	control	272	266	2	control	279	272
	p354 10-13	270	267		p356 10-13	268	275
	p354 10-12	272	267		p356 10-12	275	270
	p354 10-11	263	262		p356 10-11	282	271
	p354 10-10	258	256		p356 10-10	272	278
	p354 10-9	257	254		p356 10-9	278	273
	p354 10-8	253	252		p356 10-8	275	273
	p354 10-7	263	255		p356 10-7	277	275
	p354 10-6	254	248		p356 10-6	273	268
	p354 10-5	255	252		p356 10-5	272	297

		P4	P5			P4	P5
3	control	249	250	3	control	276	295
	p354 10-13	251	251		p356 10-13	254	244
	p354 10-12	263	255		p356 10-12	266	249
	p354 10-11	251	253		p356 10-11	256	246
	p354 10-10	266	255		p356 10-10	270	255
	p354 10-9	263	258		p356 10-9	253	248
	p354 10-8	262	257		p356 10-8	252	242
	p354 10-7	262	264		p356 10-7	253	247
	p354 10-6	260	259		p356 10-6	249	250
	p354 10-5	274	277		p356 10-5	258	356

B

		p4	p5			p4	p5
1	hKP10 10-8	340	1349	1	hKP10 10-8	432	1445
	hKP10 10-8 + P271 10-13	347	1398		hKP10 10-8 + p234 10-13	418	1329
	hKP10 10-8 + P271 10-12	361	1239		hKP10 10-8 + p234 10-12	421	1304
	hKP10 10-8 + P271 10-11	339	1330		hKP10 10-8 + p234 10-11	410	1492
	hKP10 10-8 + P271 10-10	355	1266		hKP10 10-8 + p234 10-10	430	1535
	hKP10 10-8 + P271 10-9	341	1461		hKP10 10-8 + p234 10-9	428	1392
	KP10 10-8 + P271 10-8	345	1357		hKP10 10-8 + p234 10-8	426	1532
	hKP10 10-8 + P271 10-7	345	1434		hKP10 10-8 + p234 10-7	412	1504
	hKP10 10-8 + P271 10-6	337	1547		hKP10 10-8 + p234 10-6	425	1553
	hKP10 10-8 + P271 10-5	375	1393		hKP10 10-8 + p234 10-5	424	1445
2	hKP10 10-8	422	1207	2	hKP10 10-8	426	1036
	hKP10 10-8 + P271 10-13	419	1319		hKP10 10-8 + p234 10-13	429	963
	hKP10 10-8 + P271 10-12	429	1389		hKP10 10-8 + p234 10-12	435	1030
	hKP10 10-8 + P271 10-11	416	1390		hKP10 10-8 + p234 10-11	424	1060
	hKP10 10-8 + P271 10-10	424	1329		hKP10 10-8 + p234 10-10	427	1055
	hKP10 10-8 + P271 10-9	410	1328		hKP10 10-8 + p234 10-9	424	1075
	hKP10 10-8 + P271 10-8	406	1385		hKP10 10-8 + p234 10-8	419	1089
	hKP10 10-8 + P271 10-7	475	1093		hKP10 10-8 + p234 10-7	415	1143
	hKP10 10-8 + P271 10-6	474	1114		hKP10 10-8 + p234 10-6	430	1154
	hKP10 10-8 + P271 10-5	460	1159		hKP10 10-8 + p234 10-5	425	1360

3	hKP10 10-8	449	1634
	hKP10 10-8 + P271 10-13	454	1384
	hKP10 10-8 + P271 10-12	455	1440
	hKP10 10-8 + P271 10-11	453	1250
	hKP10 10-8 + P271 10-10	452	1606
	hKP10 10-8 + P271 10-9	455	1627
	hKP10 10-8 + P271 10-8	452	1658
	hKP10 10-8 + P271 10-7	456	1657
	hKP10 10-8 + P271 10-6	449	1760
	hKP10 10-8 + P271 10-5	452	1663

3	hKP10 10-8	418	1733
	hKP10 10-8 + p234 10-13	431	1773
	hKP10 10-8 + p234 10-12	425	1863
	hKP10 10-8 + p234 10-11	417	1699
	hKP10 10-8 + p234 10-10	424	1797
	hKP10 10-8 + p234 10-9	418	1844
	hKP10 10-8 + p234 10-8	417	1814
	hKP10 10-8 + p234 10-7	408	1747
	hKP10 10-8 + p234 10-6	398	1523
	hKP10 10-8 + p234 10-5	412	1858

1	cKP10 10-8	429	2203
	cKP10 10-8 + p271 10-13	443	1988
	cKP10 10-8 + p271 10-12	438	2030
	cKP10 10-8 + p271 10-11	422	1987
	cKP10 10-8 + p271 10-10	417	1985
	cKP10 10-8 + p271 10-9	416	2018
	cKP10 10-8 + p271 10-8	411	2091
	cKP10 10-8 + p271 10-7	411	2026
	cKP10 10-8 + p271 10-6	442	1785
	cKP10 10-8 + p271 10-5	776	1052

1	cKP10 10-8	399	1938
	cKP10 10-8 + p234 10-13	392	2079
	cKP10 10-8 + p234 10-12	397	2059
	cKP10 10-8 + p234 10-11	399	2105
	cKP10 10-8 + p2340 10-10	392	2001
	cKP10 10-8 + p234 10-9	399	2062
	cKP10 10-8 + p234 10-8	400	1962
	cKP10 10-8 + p234 10-7	439	2454
	cKP10 10-8 + p234 10-6	444	2245
	cKP10 10-8 + p234 10-5	442	2323

1	hKP10 10-8	248	620
	hKP10 10-8 + p354 10-13	221	570
	hKP10 10-8 + p354 10-12	250	565
	hKP10 10-8 + p354 10-11	243	591
	hKP10 10-8 + p354 10-10	238	584
	hKP10 10-8 + p354 10-9	276	581
	hKP10 10-8 + p354 10-8	259	566
	hKP10 10-8 + p354 10-7	248	584
	hKP10 10-8 + p354 10-6	250	628
	hKP10 10-8 + p354 10-5	375	588
2	hKP10 10-8	350	894
	hKP10 10-8 + p354 10-13	329	855
	hKP10 10-8 + p354 10-12	355	845
	hKP10 10-8 + p354 10-11	348	772
	hKP10 10-8 + p354 10-10	378	760
	hKP10 10-8 + p354 10-9	337	750
	hKP10 10-8 + p354 10-8	344	767
	hKP10 10-8 + p354 10-7	338	797
	hKP10 10-8 + p354 10-6	368	810
	hKP10 10-8 + p354 10-5	552	805

1	hKP10 10-8	319	871
	hKP10 10-8 + p356 10-13	328	919
	hKP10 10-8 + p356 10-12	319	1045
	hKP10 10-8 + p356 10-11	318	1103
	hKP10 10-8 + p356 10-10	314	1067
	hKP10 10-8 + p356 10-9	310	1032
	hKP10 10-8 + p356 10-8	315	1000
	hKP10 10-8 + p356 10-7	316	1040
	hKP10 10-8 + p356 10-6	367	1111
	hKP10 10-8 + p356 10-5	671	978
2	hKP10 10-8	334	857
	hKP10 10-8 + p356 10-13	412	1007
	hKP10 10-8 + p356 10-12	348	941
	hKP10 10-8 + p356 10-11	346	925
	hKP10 10-8 + p356 10-10	327	828
	hKP10 10-8 + p356 10-9	343	872
	hKP10 10-8 + p356 10-8	317	813
	hKP10 10-8 + p356 10-7	341	647
	hKP10 10-8 + p356 10-6	385	554
	hKP10 10-8 + p356 10-5	489	534

3	hKP10 10-8	239	700
	hKP10 10-8 + p354 10-13	207	918
	hKP10 10-8 + p354 10-12	216	789
	hKP10 10-8 + p354 10-11	201	852
	hKP10 10-8 + p354 10-10	211	872
	hKP10 10-8 + p354 10-9	205	680
	hKP10 10-8 + p354 10-8	211	591
	hKP10 10-8 + p354 10-7	204	539
	hKP10 10-8 + p354 10-6	220	612
	hKP10 10-8 + p354 10-5	229	916

3	hKP10 10-8	343	1425
	hKP10 10-8 + p356 10-13	355	1371
	hKP10 10-8 + p356 10-12	330	1363
	hKP10 10-8 + p356 10-11	358	1389
	hKP10 10-8 + p356 10-10	343	1522
	hKP10 10-8 + p356 10-9	329	1443
	hKP10 10-8 + p356 10-8	347	1312
	hKP10 10-8 + p356 10-7	333	1234
	hKP10 10-8 + p356 10-6	393	1420
	hKP10 10-8 + p356 10-5	851	1383

1	cKP10 10-8	334	841
	cKP10 10-8 + p354 10-13	330	917
	cKP10 10-8 + p354 10-12	339	970
	cKP10 10-8 + p354 10-11	333	855
	cKP10 10-8 + p354 10-10	339	942
	cKP10 10-8 + p354 10-9	333	814
	cKP10 10-8 + p354 10-8	323	747
	cKP10 10-8 + p354 10-7	328	754
	cKP10 10-8 + p354 10-6	344	825
	cKP10 10-8 + p354 10-5	459	790
2	cKP10 10-8	262	700
	cKP10 10-8 + p354 10-13	305	782
	cKP10 10-8 + p354 10-12	311	802
	cKP10 10-8 + p354 10-11	310	771
	cKP10 10-8 + p354 10-10	303	762
	cKP10 10-8 + p354 10-9	307	808
	cKP10 10-8 + p354 10-8	305	682
	cKP10 10-8 + p354 10-7	306	703
	cKP10 10-8 + p354 10-6	310	676
	cKP10 10-8 + p354 10-5	322	644
3	cKP10 10-8	223	910
	cKP10 10-8 + p354 10-13	215	731
	cKP10 10-8 + p354 10-12	216	716
	cKP10 10-8 + p354 10-11	212	733
	cKP10 10-8 + p354 10-10	203	681
	cKP10 10-8 + p354 10-9	208	698
	cKP10 10-8 + p354 10-8	203	882
	cKP10 10-8 + p354 10-7	224	757
	cKP10 10-8 + p354 10-6	247	689
	cKP10 10-8 + p354 10-5	209	864

1	cKP10 10-8	319	871
	cKP10 10-8 + p356 10-13	323	980
	cKP10 10-8 + p356 10-12	316	958
	cKP10 10-8 + p356 10-11	315	967
	cKP10 10-8 + p356 10-10	307	984
	cKP10 10-8 + p356 10-9	306	878
	cKP10 10-8 + p356 10-8	316	947
	cKP10 10-8 + p356 10-7	306	895
	cKP10 10-8 + p356 10-6	342	903
	cKP10 10-8 + p356 10-5	646	833
2	cKP10 10-8	334	857
	cKP10 10-8 + p356 10-13	276	704
	cKP10 10-8 + p356 10-12	322	582
	cKP10 10-8 + p356 10-11	337	613
	cKP10 10-8 + p356 10-10	322	514
	cKP10 10-8 + p356 10-9	350	634
	cKP10 10-8 + p356 10-8	350	546
	cKP10 10-8 + p356 10-7	359	535
	cKP10 10-8 + p356 10-6	364	514
	cKP10 10-8 + p356 10-5	539	660
3	cKP10 10-8	338	1046
	cKP10 10-8 + p356 10-13	328	1103
	cKP10 10-8 + p356 10-12	338	1113
	cKP10 10-8 + p356 10-11	347	1106
	cKP10 10-8 + p356 10-10	357	1051
	cKP10 10-8 + p356 10-9	340	962
	cKP10 10-8 + p356 10-8	329	1039
	cKP10 10-8 + p356 10-7	351	1015
	cKP10 10-8 + p356 10-6	425	928
	cKP10 10-8 + p356 10-5	761	1164
