# Supplementary material for: In vitro and in vivo effects of kisspeptin antagonists p234, p271, p354, and p356 on GPR54 activation
Source: PLoS One. 2017 Jun 26;12(6):e0179156. doi: 10.1371/journal.pone.0179156 (PMC5484485; doi:10.1371/journal.pone.0179156)
Supplement: S2 Dataset — (PDF) [file pone.0179156.s002.pdf]

In vivo data: plasma LH concentrations in anestrous bitches (µg/L)

		Time (min) relative to antagonist infusion									
**peptide**	**DOG**	**-40**	**0**	**30**	**60**	**90**	**120**	**130**	**140**	**160**	**180**
p354	**a**	5,72	7,58	5,84	4,64	5,48	4,83	19,59	13,00	9,09	9,90
p354	**b**	2,77	2,77	2,06	2,21	3,03	15,74	20,29	11,26	6,21	4,54
p354	**c**	5,94	5,76	13,11	7,18	6,26	5,64	14,04	10,93	8,48	6,90
p354	**d**	13,22	5,04	4,53	3,65	3,83	4,06	13,03	9,08	6,63	4,76
p354	**e**	3,66	3,10	2,88	2,68	3,44	5,77	8,36	5,48	4,68	3,70
p354	**f**	2,96	2,99	2,34	2,58	2,74	2,57	9,96	6,75	5,03	3,82
p356	**a**	5,06	5,47	12,84	7,76	6,76	6,41	15,92	10,89	7,65	12,77
p356	**b**	5,92	3,69	3,17	2,21	3,14	2,27	23,11	13,40	7,07	4,24
p356	**c**	6,11	5,92	5,09	5,26	5,90	10,40	12,87	9,51	7,40	6,93
p356	**d**	3,14	4,56	6,79	5,41	5,79	6,70	10,88	7,82	6,69	5,19
p356	**e**	3,91	3,33	2,44	2,45	3,31	3,32	12,77	7,39	6,52	4,11
p356	**f**	2,80	2,76	2,47	2,59	2,83	2,14	9,05	7,11	4,81	3,65
p271	**a**	6,02	5,74	5,18	9,97	6,27	5,77	24,10	12,60	8,82	7,29
p271	**b**	2,71	3,54	2,64	5,88	4,15	7,50	16,64	8,91	6,48	4,68
p271	**c**	6,54	3,49	5,36	5,57	6,27	6,24	16,13	11,13	8,03	7,11
p271	**d**	4,88	4,23	4,83	4,67	4,84	6,35	9,27	7,32	5,40	4,96
p271	**g**	3,31	2,48	2,16	2,46	2,80	3,56	16,22	11,52	6,42	3,83
p271	**h**	3,51	3,20	2,99	4,88	3,89	3,20	9,44	5,71	4,76	3,51

		Time (min) relative to canine KP 10 administration					
	**DOG**	**-40**	**0**	**10**	**20**	**40**	**60**
controls	**i**	1,30	1,70	9,15	5,48	3,69	2,32
controls	**j**	2,35	1,57	7,29	4,94	2,67	2,01
controls	**k**	1,88	5,47	7,86	4,79	2,93	2,65
controls	**f**	2,00	2,34	26,83	11,88	5,24	3,46
controls	**l**	1,50	3,07	5,11	4,02	2,51	1,27
controls	**m**	1,65	1,71	10,33	7,08	4,32	2,63
